# A deep learning model adjusting for infant gender, age, height, and weight to determine whether the individual infant suit ultrasound examination of developmental dysplasia of the hip (DDH)

**DOI:** 10.3389/fped.2023.1293320

**Published:** 2023-11-16

**Authors:** Xiaoyi Chen, Shuangshuang Zhang, Wei Shi, Dechao Wu, Bingxuan Huang, Hongwei Tao, Xuezhi He, Na Xu

**Affiliations:** ^1^ Department of Ultrasound, Shenzhen Children's Hospital of China Medical University, Shenzhen, China; ^2^ Department of Orthopedics, Shenzhen Pediatrics Institute of Shantou University Medical College, Shenzhen, China; ^3^ Department of Ultrasound, Shenzhen Pediatrics Institute of Shantou University Medical College, Shenzhen, China

**Keywords:** developmental dysplasia of the hip, infant, ultrasonography, individuation, deep learning

## Abstract

**Objective:**

To examine the correlation between specific indicators and the quality of hip joint ultrasound images in infants and determine whether the individual infant suit ultrasound examination for developmental dysplasia of the hip (DDH).

**Method:**

We retrospectively selected infants aged 0–6 months, undergone ultrasound imaging of the left hip joint between September 2021 and March 2022 at Shenzhen Children’s Hospital. Using the entropy weighting method, weights were assigned to anatomical structures. Moreover, prospective data was collected from infants aged 5–11 months. The left hip joint was imaged, scored and weighted as before. The correlation between the weighted image quality scores and individual indicators were studied, with the last weighted image quality score used as the dependent variable and the individual indicators used as independent variables. A Long-short term memory (LSTM) model was used to fit the data and evaluate its effectiveness. Finally, The randomly selected images were manually measured and compared to measurements made using artificial intelligence (AI).

**Results:**

According to the entropy weight method, the weights of each anatomical structure as follows: bony rim point 0.29, lower iliac limb point 0.41, and glenoid labrum 0.30. The final weighted score for ultrasound image quality is calculated by multiplying each score by its respective weight. Infant gender, age, height, and weight were found to be significantly correlated with the final weighted score of image quality (*P* < 0.05). The LSTM fitting model had a coefficient of determination (*R*^2^) of 0.95. The intra-class correlation coefficient (ICC) for the *α* and *β* angles between manual measurement and AI measurement was 0.98 and 0.93, respectively.

**Conclusion:**

The quality of ultrasound images for infants can be influenced by the individual indicators (gender, age, height, and weight). The LSTM model showed good fitting efficiency and can help clinicians select whether the individual infant suit ultrasound examination of DDH.

## Introduction

Developmental Dysplasia of the Hip (DDH) is a common type of bone and joint diseases among infants, including hip dislocation, subluxation, dysplasia with or without instability and other subtypes (1). The incidence of this disease varies significantly among different ethnic groups and countries and regions, ranging from 0.15%–2% (2). It has been established that early diagnosis of DDH can lower the need for surgery, prevent complications, and improve the prognosis for infants.

Common techniques used for examining Developmental Dysplasia of the Hip (DDH) include ultrasound, x-ray, CT, MRI, and others. Currently, ultrasound and x-ray are the most commonly used methods for screening and diagnosing DDH (3). Ultrasound is the preferred imaging method for infants under 6 months of age because secondary ossification centers typically develop between 3 and 6 months. Furthermore, ultrasound provides optimal visualization of the hip before the formation of femoral head ossification centers (4). When the femoral head’s ossification center starts to develop, it can make it difficult to see the lower iliac limb point in ultrasound imaging. Therefore, for the diagnosis of DDH in infants older than six months, x-ray is often used (3). CT is better suited to display the bone cortical structure and is therefore used (5). Magnetic resonance technology has excellent soft tissue resolution and can be used for three-dimensional evaluation, making it an important auxiliary tool in the diagnosis and treatment of DDH. Each type of imaging test has its own advantages and disadvantages. For example, there is radiation exposure when using x-rays and their accuracy can be affected by the position of the child, resulting in false positives and false negatives (6). CT scans make child more radiation exposure and cannot show soft tissue structure, so they are often used for pre-surgical evaluation. MRI scans are very expensive and require sedation, making them inconvenient. Compared to other imaging tests, ultrasound examinations are safer and more reliable. However, the accuracy of ultrasound imaging diagnosis depends on the quality of the images produced.

In clinical practice, it has been found that the quality of hip joint ultrasound images in some young infants is not optimal, while some older infants’ ultrasound images can clearly display standard sectional anatomy and thus obtain accurate ultrasound diagnosis. Therefore, age alone cannot be used as the sole basis for ultrasound examination of DDH, as this may be related to individual development such as infant age, height, weight, and other factors.

In this research, we assessed the factors that affect the quality of ultrasound images in infants by considering their gender, age, height, and weight. We objectively assigned weights to the evaluation scores of the images using the entropy weight method and used a deep learning model-LSTM to develop the model. Long-short term memory network (LSTM) is a type of neural network that possesses temporal prediction characteristics (7). It comprises three gate structures, namely, forgetting gate, input gate, and output gate. It can extract and analyze various data characteristics, enabling dynamic time-point data analysis and prediction. Our goal was to investigate the suitability of ultrasound examinations for diagnosing DDH among different individuals and provide guidance for selecting the ultrasound examination.

## Methods

### Study population

**Figure 1:**
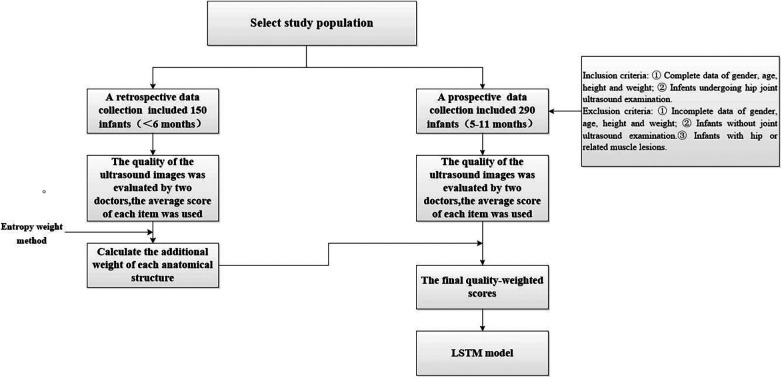
Flowchart of the present study.

Between September 2021 and March 2022, a retrospective data collection was conducted at Shenzhen Children’s Hospital. We included 150 infants, comprising of both 77 male and 73 female infants, aged between 0 and 6 months. All infants received hip joint ultrasound examination during this period and were included in the study for entropy weight calculation. Inclusion criteria as following: infants with complete individual developmental indicator data for gender, age, height, and weight, who have undergone hip joint ultrasound examination. Exclusion criteria were infants with incomplete individual developmental indicator data for gender, age, height, and weight, who have not undergone hip joint ultrasound examination, and infants with hip joint or related muscle lesions. From July 2022 to December 2022, a prospective collection of data was conducted at the Ultrasound Department of Shenzhen Children’s Hospital. The data involved 290 infants between the ages of 5 and 11 months who underwent hip joint ultrasound examination, including both 153 male and 137 female infants. Specifically, there were 50 cases each for infants aged 5, 6, 7, and 8 months, and 30 cases each for infants aged 9, 10, and 11 months. This study was approved by the Ethics Committee of Shenzhen Children’s Hospital (approval number: 2022078). All patients were informed of the examination method and purpose and signed informed consent prior to examination. The participants of the study were requested to provide their personal information, including gender, age, height, and weight, on the day of their examination. The individual indexes of these demographic variables were recorded for each subject as part of the data collection process (Figure 1).

### Ultrasound examination

**Figure 2:**
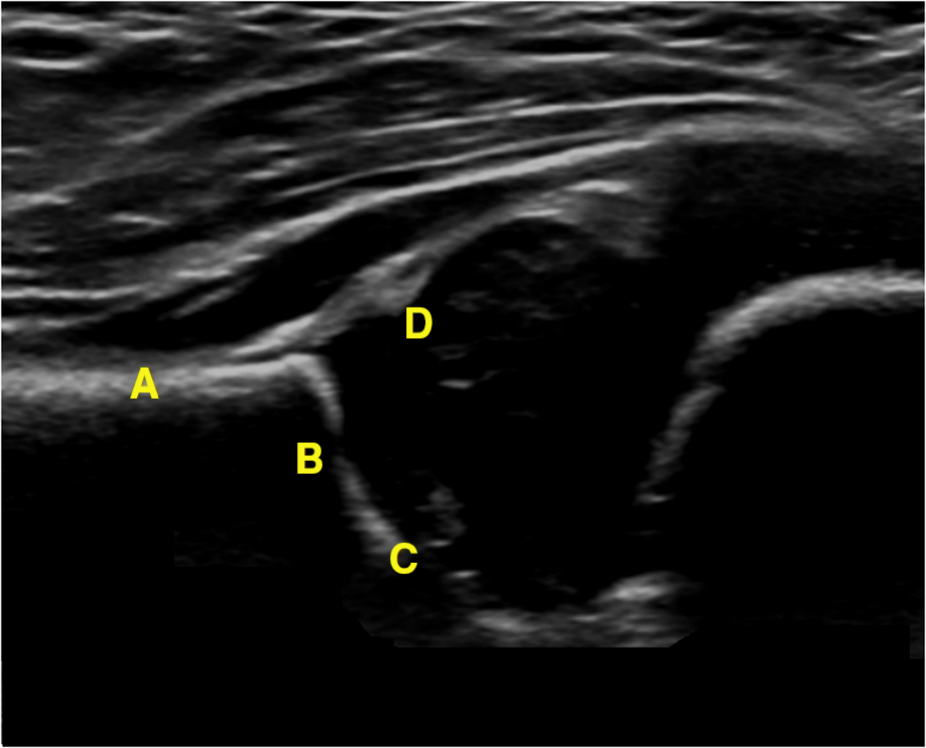
Measurement of the neutral coronal plane of the hip joint using the graf method. (**A**) Iliac bone, (**B**) bony rim point, (**C**) lower iliac limb point, (**D**) glenoid labrum.

In this study, the GE LOGIQ-E9 ultrasonic diagnostic instrument (America) and 9l linear probe with a frequency range of 5.0–9.0 MHz were employed for imaging of the hip joint. The ultrasound coronal plane of Graf’s method was chosen as the standard imaging technique for this purpose. The imaging procedures were conducted by experienced senior physicians who possess the necessary expertise and technical skills in ultrasound imaging. In the standard imaging, four markers were identified, namely the iliac bone, bony rim point, lower iliac limb point, and glenoid labrum, as illustrated in Figure 2. These markers were selected based on their ability to provide accurate and reliable measurements of the hip joint. The utilization of a standardized imaging protocol and the identification of these markers allowed for consistent and reproducible imaging of the hip joint, ensuring the validity and reliability of the data obtained in this study.

### Score of ultrasound image quality

In this study, considering there was no relationship between straightness of iliac bone and individual indicators such as weight and age in children, three anatomical structures were included to evaluate image quality. The quality of the ultrasound images was evaluated by two doctors based on the clarity of the three critical anatomical structures displayed in the hip joint ultrasound images, as outlined in Tables 1, 2. Each structural point was assigned a score ranging from 1 to 5, with a score of 3 or higher indicating that the image was of sufficient quality for accurate measurements to be taken. By employing these standardized scoring criteria, the experienced doctors were able to assess the quality of the ultrasound images objectively and consistently, ensuring that the images used in subsequent analyses were of sufficient quality and accuracy.

**Table 1:** Image quality scores.

Score	Bony rim point	Lower iliac limb point	Glenoid labrum
1	Not shown	Not shown	Not shown
2	Unclear	Unclear	Unclear
3	Measurable	Measurable	Visible
4	Visible	Visible	Clearness
5	Clearness	Clearness	Clearness and regular

**Table 2:** Image quality scores (image plate).

Score	Bony rim point	Lower iliac limb point	Glenoid labrum
1	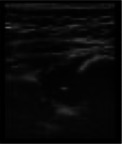	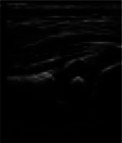	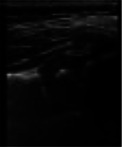
2	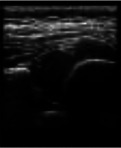	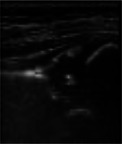	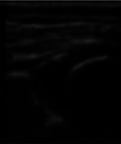
3	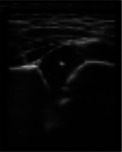	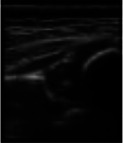	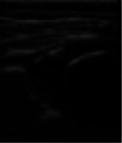
4	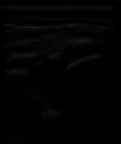	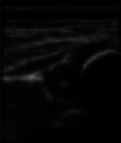	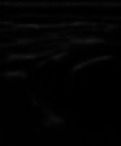
5	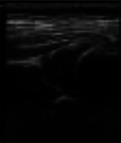	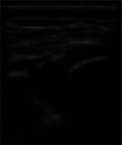	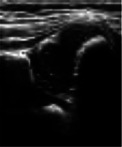

### Assigning weights with entropy weight method

150 ultrasound images of hip joints were retrospectively selected. Two doctors scored the quality of ultrasound images according to the clarity of the three key anatomical structure displayed in the ultrasound images of hip joints (Tables 1, 2). Each structural point is scored with a total score of 5, 3 or above was qualified, indicating that the image can be measured. All images were scored by two senior doctors, and the average score of each item was used to calculate the additional weight of each anatomical structure by entropy weight method, as seen in Appendix.

A total of 290 prospective ultrasound images of the left hip joint of the subjects were manually scored. The entropy weight method was used to calculate weights for each anatomical structure, and the final quality-weighted scores for all 290 images were obtained. A final ultrasonic image quality score greater than 3 points indicates clear and measurable image structure, allowing for accurate diagnosis.

### Consistency of manual and AI measurement of *α* angles and *β* angles

15 cases (>6 months) were randomly selected from ultrasonic images whose final score was greater than 3 points for manual and AI automatic measurement of *α* Angles and *β* Angles, and recorded the measured angles for consistency test.

### Statistical methods

**Figure 3:**
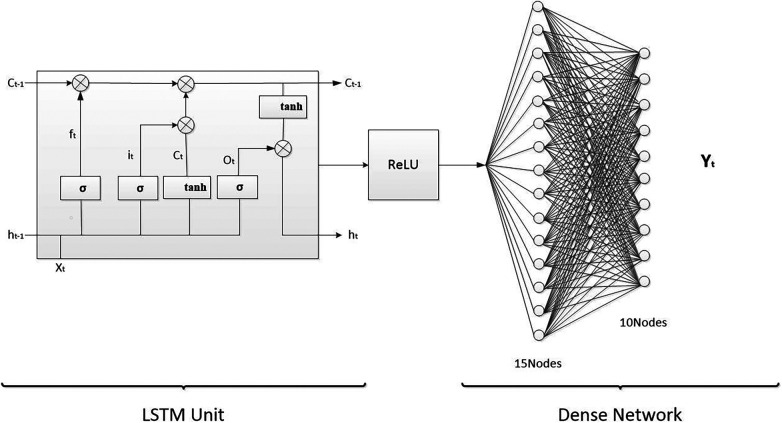
Schematic diagram of the LSTM fitting model.

Statistical analysis was performed using SPSS 26.0 software. Normally distributed data were presented as mean ± SEM. The intra-class correlation coefficient (ICC) was used to investigate the correlation between the anatomical structure scores of each ultrasound image in the two groups. Pearson correlation analysis was used to assess the association of individual indices such as height and weight, with the image quality score. Independent sample *t*-test was conducted for comparison of the image quality scores between male group and female group. The image quality scores were also grouped according to month age, and ANOVA was performed for analysis. The ICC was used to test the correlation between manual measurement and AI measurement of *α* Angles and *β* Angles. A *P* value of less than 0.05 was considered statistically significant. An LSTM model was constructed using Matlab software with 240 training sets and 50 test sets (Figure 3). The closer the determination coefficient of the fitting model is to 1.0, the higher the fitting efficiency. Conversely, the closer it is to zero, the less efficient it is.

## Result

### Ultrasonic image quality score

A total of 50 ultrasound images of the left hip joint, which were less than 6 months old, were retrospectively collected and assessed for four anatomical structures. The scores of Doctor 1 for bony rim point, lower iliac limb point, and glenoid labrum were respectively 3.17 ± 1.13, 3.27 ± 1.35, and 2.52 ± 1.03. Similarly, Doctor 2’s scores for the same structures were 2.99 ± 0.99, 3.21 ± 1.23, and 2.47 ± 0.85, respectively. The ICC values for the scores of bony rim point, lower iliac limb point, and glenoid labrum in hip joint ultrasound images were 0.78, 0.88, and 0.79, respectively (Table 3). Both doctors’ scores showed good consistency, and the average score for each item was used in the analysis.

**Table 3:** Consistency test of two doctors’ image scores.

Group	Bony rim point score	Lower iliac limb point score	Glenoid labrum score
Doctor 1	3.17 ± 1.13	3.27 ± 1.35	2.52 ± 1.03
Doctor 2	2.99 ± 0.99	3.21 ± 1.23	2.47 ± 0.85
ICC	0.78	0.88	0.79

### Weight of each anatomical structure of hip joint

**Figure 4:**
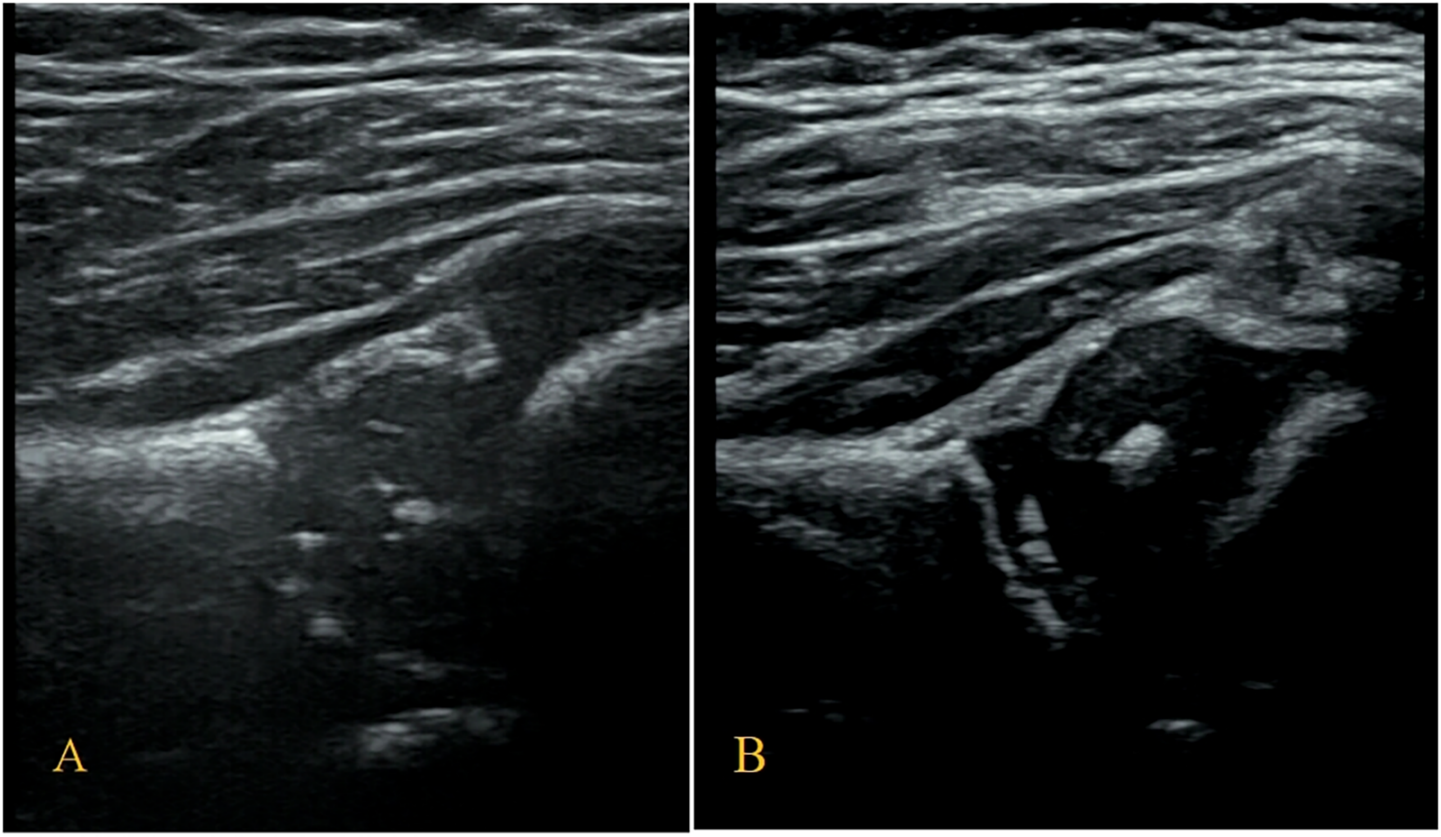
(**A**) The image structure of the 5-month-old subject was not clearly displayed, scoring only 2.30. (**B**) In contrast, the 8-month-old subjects had a clearly displayed image structure, achieving a score of 4.01.

In this study, the weights of each anatomical structure obtained by entropy weight method were respectively bony rim point: 0.29, lower iliac limb point: 0.41, glenoid labrum: 0.30. That means the last weighted score of ultrasound image quality = bony rim point × 0.29 + lower iliac limb point × 0.41 + glenoid labrum × 0.30 (Figure 4). And the proportion of the final weighted score of ultrasound image lower than 3 in each month were 16%, 20%, 14%, 14%, 33%, 27%, 43%.

### Individual index

The participants’ height, weight, and image quality scores followed a normal distribution. Height and weight were found to be negatively correlated with the weighted scores of the final image quality, with respective correlation coefficients (R) of −0.16 and −0.59 (*P* < 0.05). The male and female groups had weighted scores of 3.22 ± 0.59 and 3.37 ± 0.58, respectively, with a statistically significant difference (*P* < 0.05). The final image quality’s weighted scores were grouped by the participants’ age in months. ANOVA analysis showed statistically significant differences in age in months.

### LSTM model fitting of individual index and final image quality weighted fraction

**Figure 5:**
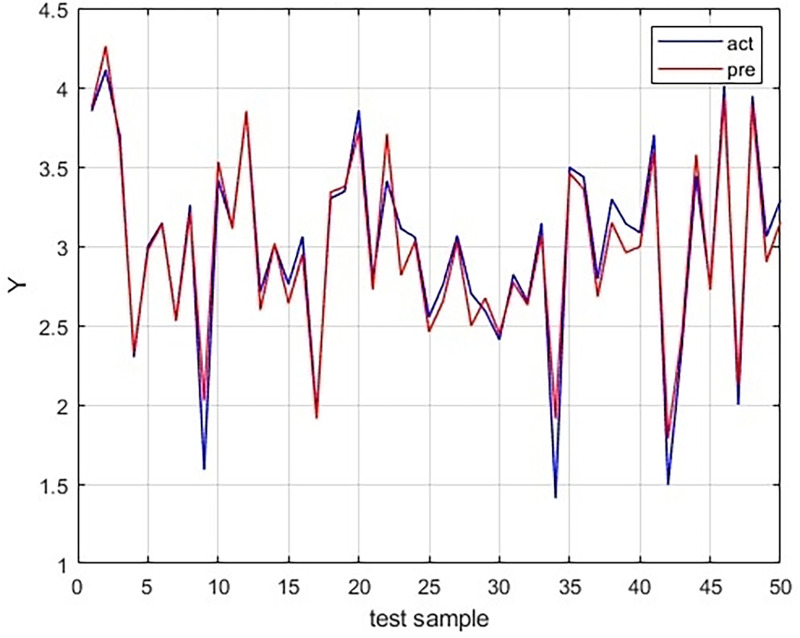
Comparison of the predicted values using LSTM model with the actual values in the testing dataset.

**Figure 6:**
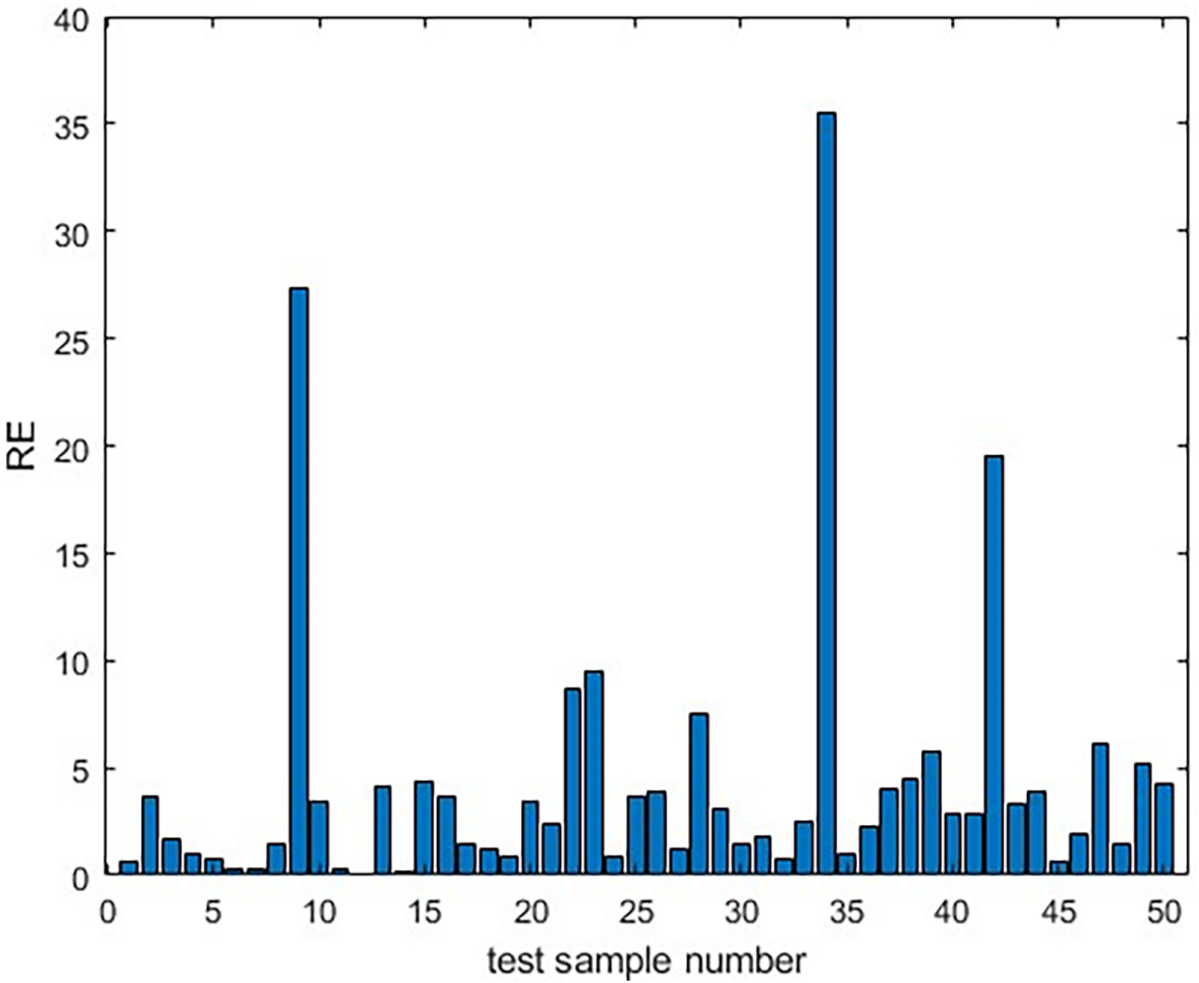
The relative error between the predicted values and the actual values of the LSTM model in the testing dataset.

The fitted model of LSTM for the test set shows a good match between the predicted and actual values, as seen in Figure 5. The relative errors were almost below 10%, as depicted in Figure 6.

### Comparison of consistency of manual and AI measurement

Fifteen cases older than 7 months with image quality scores greater than 3 points were randomly selected for manual and AI measurement of *α* Angles and *β* Angles. ICC test was performed and the intra-group consistency were 0.98 (*α* Angles group) and 0.93 (*β* Angles group), respectively (Table 4).

**Table 4:** Consistency inspection of *α* angle and *β* angle between manual measurement and AI measurement.

Group	*α* Angles	*β* Angles
Manual measurement	69.27 ± 3.53	46.80 ± 2.24
AI measurement	69.33 ± 3.99	47.00 ± 2.95
ICC	0.98	0.93

## Discussion

To determine whether a section of DDH ultrasonic image is standard and whether accurate ultrasonic diagnosis can be performed, there are several studies available that assess the quality of DDH ultrasonic images. Hongmei Dong et al. (8) evaluated ultrasonic images of DDH and conducted quality control on three anatomical structures: the iliac bone, lower iliac limb point, and glenoid labrum. During the evaluation, the investigators considered various factors such as the image gain, the drawing line used during measurement, and the size of the image. Abhilash et al. (9) employed a 10-point scale to assess various factors, including the ilium, labrum, os ischium, femoral head, motion artifact, and other imaging artifacts, such as limited penetration or excessive image noise. The scores assigned to each indicator evaluation were 2, 1, 2, 1, 2, and 2, respectively, to artificially assign weights to each indicator. However, both of these evaluation methods are inevitably subjective. In this study, we excluded other evaluation indicators, such as drawing lines and image size, that would not affect the diagnosis. To reduce human subjectivity in the evaluation process, we introduced the entropy weight method to objectively assign weight to scores.

The entropy weight method is a scientific approach to decision-making that evaluates indicators’ objective weighting comprehensively based on the information contained in the evaluation objects (10). This method can decrease the subjectivity of artificial ratings and is extensively employed in various fields such as chemistry, architecture, and engineering technology. It is mostly used in public health and infectious diseases, but its usage in the medical sector is limited (11, 12). In this study, the final image quality score, calculated using the entropy weight method, is more objective and reliable than direct human scoring and artificial weighting.

According to the present guidelines, ultrasound screening can be selected for infants who are younger than 6 months (1). This is because after 6 months, the role of ultrasound becomes limited due to the formation of the secondary ossification center of the femoral head, which blocks the deep structures of the hip joint. At this stage, x-ray diagnosis is recommended instead. However, it’s important to note that the 6-month threshold is a broad boundary. Previous studies have demonstrated that high-quality ultrasound images can be acquired, enabling accurate diagnoses even for infants with secondary ossification centers in later months (13). In infants older than 8 months, the proportion of image quality scores under 3 points increase significantly. Our results showed that for older infants, particularly those aged 7–8 months, the small ossification center may not obstruct the lower iliac limb point, allowing for precise ultrasound diagnosis of DDH in these cases. Aside from age, infant growth and development rates differ among individuals, which can impact ultrasound examination results. Therefore, it is not reasonable to solely rely on age as a determinant for examination methods. The aim of this study is to establish a dependable foundation for making ultrasound decisions based on an individual’s unique characteristics.

Several individual indicators have been found to affect the quality of two-dimensional ultrasound images, and numerous scholars have conducted related research. Several studies have confirmed that body mass index has a significant impact on ultrasound image quality, particularly for abdominal examinations (14–16). This is because the abdomen tends to accumulate fat. Likewise, when examining children’s hip joints, the probe is typically positioned at areas with high fat concentration, which can also affect image quality. Additionally, there are differences in growth and development between male and female infants. Therefore, this paper examines the correlation between infants’ individual indexes (gender, age, height, and weight) and ultrasound image quality, and concludes that gender, age in months, height, and weight all have an impact on the ultrasound image quality score.

Currently, LSTM is mainly used in the form of a composite network, connected with a Convolutional Neural Networks (CNN) network, for disease diagnosis, differential diagnosis, risk assessment, and classification of ultrasound images, among others (17–20).As the data of infant age is in the form of a time series, an LSTM model was employed in this study. The final image score, which was calculated using the entropy weight method, was used as the dependent variable, while infant gender, age, height, and weight were used as the independent variables for model fitting.

Enter the infant’s gender, age, height, and weight to predict the quality score of its ultrasound image. The *R*^2^ regression model has achieved a score of 0.95, which can accurately estimate whether children are suitable for an ultrasound examination, providing clinicians with a basis for conducting the examination. Ultrasound is an economical, quick, and radiation-free method, and this model provides a better option for screening older children’s hip joints, making parents more comfortable with accepting ultrasound. Subsequently, 15 images featuring subjects with ultrasonic image scores greater than 3 were randomly chosen for automatic scanning using AI (21). The obtained measured values exhibited a high degree of consistency with those that were manually measured, thus indicating that the anatomical structure of the images analyzed in this study was clear, accurate, and diagnosable. This paper serves as further evidence of the efficacy of this image quality scoring method.

In this study, the number of older subjects was limited, and the method of image quality assessment was restricted to specific anatomical structures, without considering the overall image quality. However, with the advancement of science and technology, the penetration of ultrasonic instruments is increasing, which will lead to an improvement in image quality. In the future, if the sample size is expanded and objective evaluation values of the overall ultrasonic image are added, using computer technology such as gray mapping function, image contrast, contrast-to-noise ratio (CNR), and high contrast spatial resolution (22), it can avoid subtle visual differences caused by advanced mode and image differences caused by different machines. This will make the score more objective and truthful, thereby improving the accuracy of the assessment.

## Conclusion

The quality of ultrasound images for infants can be influenced by their gender, age, height, and weight. However, high-quality hip joint ultrasound images can still be reliably measured by both manual and AI methods, with consistent results for *α* and *β* angles above three anatomical structures: bony rim point, lower iliac limb point, and glenoid labrum. This allows for a good evaluation of ultrasound image quality. The LSTM model has also shown good fitting efficiency, which can help guide clinicians in determining whether the individual infant suit ultrasound examination of DDH. This can help reduce the waste of medical resources and avoid the radiation exposure associated with x-ray examinations.

## Data Availability

The datasets generated and/or analysed during the current study are not publicly available due to regulation of Shenzhen Children’s Hospital and protecting patient personal information. Requests to access these datasets should be directed to the corresponding author (Na Xu), 46911069@qq.com.

## Appendix

The process of image evaluation model based on entropy weight method as follows:

(1) According to the anatomy structure score of each image, a scoring matrix of anatomical structure indicators for each image is obtained, with *m* images and *n* indicators, *X_ij_* represents the value of the *j*-th indicator of image *i*.
(2) Equation for calculating the weight of image i in the *j*-th index:
  $$P_{\mathrm{ij}}=\frac{X_{ij}}{\sum_{i=1}^{n}X_{ij}}$$
(3) Equation for calculating the entropy value of the *j*-th index, of note, *q* < 0, $q=\frac{1}{\ln(n)},e_{j}>0$, and *n* is the number of images in the following equation:
  $$e_{j}=-q\overset{n}{\underset{i=1}{\sum }}\ln(P_{ij})\;\;(i=1,\;2,\;\ldots ,n\;\;j=1,2,\ldots ,m)$$
(4) Calculate the difference coefficient of the *j*-th index. For the *j*-th index, the greater the difference between index values, the greater the left and right sides of image evaluation, and the smaller the entropy value. The difference coefficient is defined as follows:
  $$g_{j}=1-e_{j}\;\;(\;j=1,2,\ldots ,m)$$

In the formula $0\leq g_{j}\leq 1$.

(5) To find the weight coefficient of the *j*-th index:
  $$w_{j}=\frac{g_{j}}{\sum_{\;j=1}^{m}g_{j}}\;\;(\;j=1,2,\ldots ,m)$$
